# Novel design principles enable specific targeting of imaging and therapeutic agents to necrotic domains in breast tumors

**DOI:** 10.1186/bcr2579

**Published:** 2010-05-24

**Authors:** Liat Goldshaid, Efrat Rubinstein, Alexander Brandis, Dadi Segal, Noa Leshem, Ori Brenner, Vyacheslav Kalchenko, Doron Eren, Tamar Yecheskel, Yoseph Salitra, Yoram Salomon, Avigdor Scherz

**Affiliations:** 1 Department of Plant Sciences, Weizmann Institute of Science, Herzel Street, Rehovot, 76100, Israel; 2 Department of Veterinary Resources, Weizmann Institute of Science, Herzel Street, Rehovot, 76100, Israel; 3 Steba Laboratories, Ltd., Einstein Street, Kiryat Weizmann Science Park, Rehovot, 76470, Israel; 4 Department of Biological Regulation, Weizmann Institute of Science, Herzel Street, Rehovot, 76100, Israel

## Abstract

**Introduction:**

Necrosis at the tumor center is a common feature of aggressive breast cancers and has been associated with poor prognosis. It is commonly identified by means of invasive histopathology, which often correlates with morbidity and potential tumor cell dissemination, and limits the reconstruction of the whole necrotic domain. In this study we hypothesized that non covalent association to serum albumin (SA) and covalent binding to ligands for tumor-abundant cell receptors should synergistically drive selective accumulation and prolonged retention of imaging and therapeutic agents in breast tumor necrotic domains enabling *in vivo *identification, imaging and possibly treatment of such tumors.

**Methods:**

Cyclo-Arg-Gly-Asp-D-Phe-Lys (c(RGDfK)) were conjugated to bacteriochlorophyll-derivatives (Bchl-Ds), previously developed as photodynamic agents, fluorescent probes and metal chelators in our lab. The c(RGDfK) component drives ligation to α_V_β_3 _integrin receptors over-expressed by tumor cells and neo-vessels, and the Bchl-D component associates to SA in a non-covalent manner. STL-6014, a c(RGDfK)-Bchl-D representative, was i.v. injected to CD-1, nude female mice bearing necrotic and non-necrotic human MDA-MB-231-RFP breast cancer tumors. The fluorescence signals of the Bchl-Ds and RFP were monitored over days after treatment, by quantitative whole body imaging and excised tumor/tissue samples derived thereof. Complementary experiments included competitive inhibition of STL-6014 uptake by free c(RGDfK), comparative pharmacokinetics of nonconjugated c(RGDfK) Bchl-D (STL-7012) and of two human serum albumin (HSA) conjugates: HSA-STL-7012 and HSA-STL-6014.

**Results:**

STL-6014 and STL-7012 formed complexes with HSA (HSA/STL-6014, HSA/STL-7012). STL-6014, HSA-STL-7012 and HSA-STL-6014, selectively accumulated at similar rates, in tumor viable regions over the first 8 h post administration. They then migrated into the necrotic tumor domain and presented tumor half lifetimes (T_1/2_) in the range of days where T_1/2 _for HSA-STL-6014 > STL-6014 > HSA-STL-7012. No accumulation of STL-7012 was observed. Pre-injection of c(RGDfK) excess, prevented the uptake of STL-6014 in the small, but not in the large tumors.

**Conclusions:**

Non-covalent association to SA and covalent binding to c(RGDfK), synergistically enable the accumulation and prolonged retention of Bchl-Ds in the necrotic regions of tumors. These findings provide novel guidelines and strategy for imaging and treatment of necrotic tumors.

## Introduction

Upon outgrowth of their oxygen supply, emerging tumors develop hypoxic and eventually necrotic regions believed to be the direct result of chronic ischemia caused by vascular collapse when the rate of tumor cell growth exceeds that of angiogenesis [1]. Initially, necrotic areas within solid tumors constitute distinct morphologically identifiable regions that contain highly eosinophilic cells. Subsequently, this pattern is replaced by liquefaction necrosis, in which the breakdown of cellular structures can be observed [1]. Necrosis in invasive carcinoma of the breast has been correlated with the concomitant angiogenesis, development of high vascular density, and increased levels of focal macrophage infiltration driven by chemotactic factors [1-4].

Assessment of the degree of necrosis has been proposed to serve as a powerful prognostic marker for different cancer types [5-7]. Necrosis at the tumor center is a common feature of aggressive breast cancer and has been associated with poor prognosis. In particular, large necrotic volumes in ductal carcinoma *in situ *(DCIS) demonstrate tight correlation with comedo as opposed to non-comedo DCIS [8]. Necrosis in this tumor type was shown to be associated with hypoxia [4], where prolonged hypoxic conditions were found to increase the rates of mutations, tumor progression and angiogenesis, and to promote metastatic potential and pre-growth signaling pathways. In summary, the magnitude of necrosis in mammary ducts appears to positively correlate and possibly predict DCIS aggressiveness [9]. Necrotic and hypoxic conditions constitute major impediments to successful cancer therapy [10]. Therefore, increased detective resolution of hypoxic and necrotic tumors *in vivo *will enhance diagnostic procedures and better outline the tumor status for selection of appropriate treatment strategies [11-14].

Necrosis and hypoxia are commonly identified by means of invasive histopathology, which is often correlated with morbidity and potential tumor cell dissemination. Thus, *in situ *diagnostic techniques, such as magnetic resonance imaging (MRI) [15,16], blood oxygenation level dependent-MRI [17], diffusion-weighted MRI [18] and positron emission tomography (PET) [19] can be advantageous. However, insufficient vascularization, linked with enhanced interstitial fluid pressure due to the lack of lymphatic drainage, interfere with the convectional uptake of small molecules used as therapeutic or contrast agents in such detection protocols. At the same time, enhanced tumor vascular permeability in these regions drives extravasation of macromolecules such as serum albumin (SA) from the circulation into the tumor tissue, while the poor lymphatic drainage fosters their retention within the tumor compartment [20,21]. Consequently, the 'enhanced permeability and retention effect' (EPR), has been proposed as the basis for nonspecific targeting of drugs comprising large molecules to tumor tissue and has given rise to a new approach for tumor-targeting drug design based on macromolecular, micellar and lipidic particles (for a recent review see Maeda and colleagues [22]). For example, non-covalent association of styrene-maleic acid copolymer-conjugated neocarzinostatin (16 kDa) with albumin, provides about an 80 kDa complex that accumulates and exhibits retention in tumors of different origins [22]. Similarly, covalent conjugation of the therapeutic agents mitomycin C-bovine or indotricarbocyanine (ITTC) to SA, resulted in their delivery to and accumulation/retention in tumors and were even detected in the tumor necrotic domains [23]. Gadophrin-2A, a porphyrin-based necrosis avid contrast agents (NACA) used as an MRI contrast enhancing agent in necrotic tumor imaging, has been proposed to accumulate in the margins of HT29 human colon tumor necrotic zones, following delivery as a non-covalent complex with SA either as is or after dissociation in the tumor necrotic domain [24].

Recent studies showed better accumulation of NACA, which have been conjugated to large molecules that function as ligands to over-expressed tumor cell receptors. Thus, the ITTC accumulation in HT29 human colon tumors was improved after its covalent conjugation to transferrin in place of albumin, presumably because of the transferrin interactions with the cancer cell surface receptors [25]. However, such high molecular weight complexes may elicit an immune response that should prevent their frequent application as needed for imaging and therapeutic applications. Thus, despite the advances made in tumor-targeting techniques there is a clear need to recruit new imaging principles and to expedite the development of novel agents for such purposes.

In our quest for new imaging avenues, we postulated that small contrast and/or therapeutic agents designed to have the dual capacity of moderate association affinity to SA and high affinity to tumor-specific receptors, would allow for their prolonged accumulation in necrotic tumor domains. We assumed that the EPR effect would assist agent extravasation and retention in the tumor interstitium upon forming a complex with SA. Once in the tumor tissue, the modified agents will dissociate from the SA and preferentially bind to specific receptors, ensuring active agent accumulation and possibly migration to the necrotic domain by means of diffusion. Notably, the design principle of such non-covalent complexes with SA is profoundly different from the one underlying the make up of covalent SA complexes [26].

**Figure 1:**
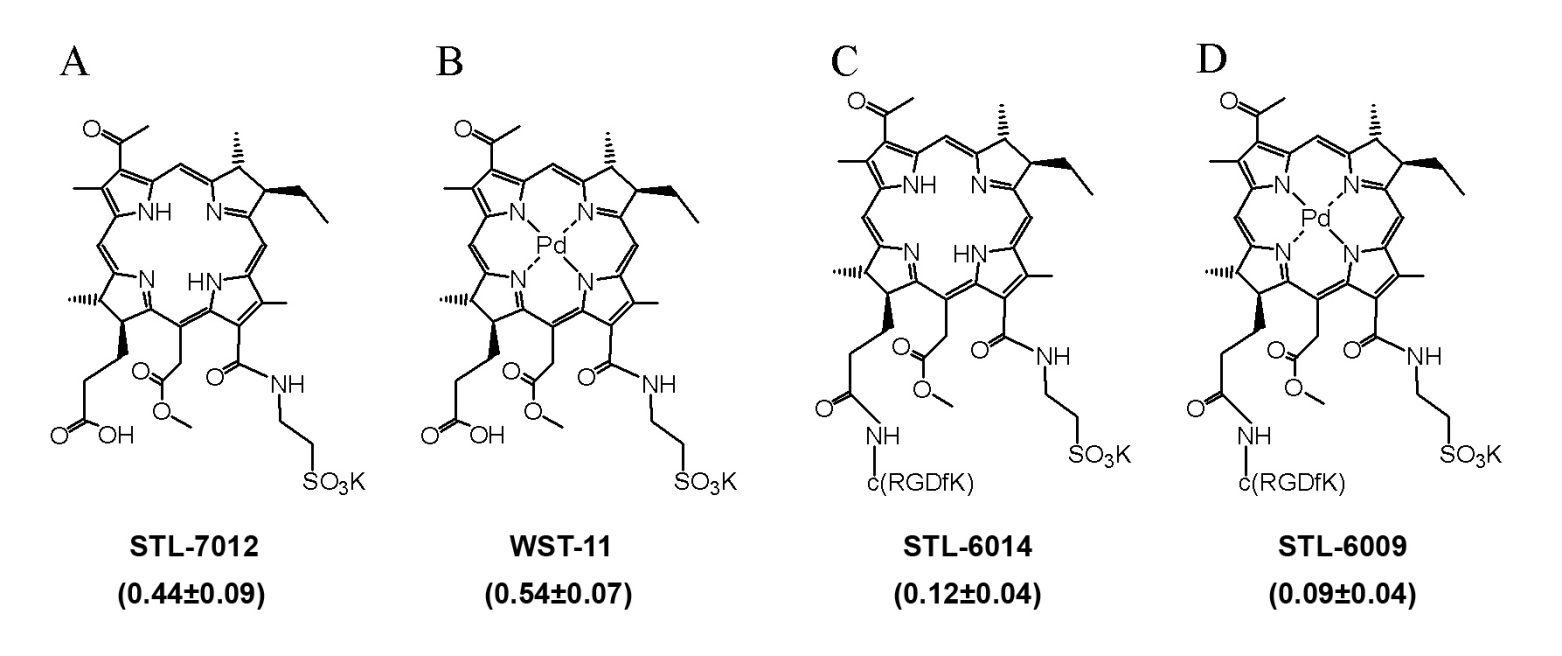
**Structures of Bchl-Ds**. Values in parenthesis are the HSA association constants Ka_(HSA)_, of each bacteriochlorophyll derivative (Bchl-D; 1/μM).

In previous and ongoing studies, we have shown that bacteriochlorophyll derivatives (Bchl-Ds) can be used as therapeutic agents in photodynamic therapy by generating oxygen radicals upon *in situ *illumination [27-35]. WST11 and other Bchl-Ds were found to be highly effective in vascular-targeted photodynamic therapy in both preclinical studies of different solid tumor types [27-32] and in the clinical studies of prostate cancer [32-35]. In addition, chemical modifications of the Bchl-D center, for example by metal or proton substitution, render them effective contrast agents for MRI [36], PET scan [37], and fluorescence imaging protocols (described below). Water-soluble Bchl-Ds, such as WST11 (Figure 1), circulate as non-covalent complexes with SA until clearance, but despite this association they show no accumulation in the tumor tissue and rapidly clear from the treated subject [28,38-40]. Thus, by covalent binding of Bchl-Ds to tumor-specific ligands while retaining their SA complexation ability, we are expecting to obtain contrast and therapeutic agents that selectively accumulate in the tumor necrotic domains, following the above working hypothesis.

Herein, we examined the application of cyclic compounds containing the Arg-Gly-Asp (RGD) sequence as to impart tumor-specific ligands for targeting Bchl-Ds/SA to necrotic tumor domains. The RGD motif is recognized by many members of the integrin family [41]. More specifically, c(RGDfK), and similar compounds demonstrated significant affinity to αVβ3 integrin receptors that are abundant in several cancer cell lines and in the rapidly developing neo-vasculaturization near necrotic tumor domains. Hence, RGD-containing molecules have been suggested to provide new opportunities for selective tumor targeting of imaging and therapeutic agents [42]. One of the problems with such application is the short life-time of the small RGD complexes in the circulation (two to three minutes lifetimes for c(RGDfK) [43]. The covalent binding of cyclic RGD to macromolecules such as human SA (HSA), results in a better accumulation of the cyclic RGD compound in tumors probably because of the significantly enhanced life time of the RGD-HSA complex in the circulation compared with the free RGD [26] and the involvement of the aforementioned EPR effect. However, as noted above the resulted protein complex may be immunogenic and therefore of limited use in the clinical arena. Hence, mobilization of the therapeutic or contrast agents by reversible, non-covalent association *in situ *with circulating SA, as proposed in the present study, is expected to be advantageous.

Recently, we synthesized and tested the uptake by tumors of STL-6009, a c(RGDfK)-conjugated WST11 (Figure 1, [37,44]). The compound selectively accumulated to significant concentrations (4 to 8 μM) in primary xenografts in mice. These concentrations mirrored the circulating C_max _values recorded for nonconjugated WST11 using inductively coupled plasma mass spectroscopy measurements. STL-6009 accumulation exhibited tight dependence on the presence of αVβ3 receptors in the respective tumor cell lines and on the extent of neovascularization in the xenograft model [37]. However, the weak fluorescence of STL-6009 due to the central palladium (Pd) atom [45], impaired the clarity of on-line fluorescence imaging, rendering its further development for imaging purposes futile. Thus, in the present study the central Pd atom was substituted by two protons yielding STL-6014 (Figure 1), a novel RGD-Bchl-D conjugate that emits 100-fold stronger fluorescence than its Pd-chelated counterpart. STL-6014 uptake was monitored in mice bearing MDA-MB-231-red fluorescence protein (RFP) breast tumors with and without central necrosis and compared with that of the following compounds: (1) STL-7012, an RGD-free analog; (2) HSA-STL-6014; and (3) HSA-STL-7012. The experimental findings combined with some literature data strongly support the above suggested hypothesis. Namely, non-covalent association to SA, and covalent binding to ligands of tumor-abundant cell receptors, synergistically drive selective accumulation and prolonged retention of agents in the necrotic domains of breast tumors, enabling the *in vivo *imaging of such necrotic tumors.

## Materials and methods

### Materials

#### Bchl-D preparation

Bchl-Ds including 3^1^-oxo-15-methoxycarbonylmethyl-Rhodobacteriochlorin 13^1^-(2-sulfoethyl)amide dipotassium salt (STL-7012, Figure 1a), and its palladium complex (WST11, Figure 1b), 3^1^-oxo-15-methoxycarbonylmethyl-Rhodobacteriochlorin 13^1^-(2-sulfoethyl)amide-17^3^-c(RGDfK)amide potassium salt (STL-6014, Figure 1c), and its palladium complex (STL-6009, Figure 1c), were prepared as previously described [37,38]. The Bchl-Ds were dissolved in 5% aqueous mannitol and adjusted to a pH of 7.2 to 7.4 with Tris HCl (10 mM tris(hydroxymethyl) aminomethane in 5% aqueous mannitol). The obtained solutions were filtered through 0.2 μm polytetrafluoroethylene (PTFE) filters (National Scientific, Rockwood Tennessee, USA). Concentrations were spectrophotometrically determined in methanol at 747 nm, using molar extinction coefficients of 1.2 × 10^5 ^M^-1^cm^-1 ^for Pd-containing and 6.3 × 10^4 ^M^-1^cm^-1 ^for Pd-free compounds. The solutions were stored in the dark at -20°C until use.

#### HSA-STL-6014 and HSA-STL-7012 preparation

c(RGDfK)-bacteriopheophorbide *a *(Bpheid) was synthesized via activation of Bpheid [38] with N-hydroxysuccinimide (NHS) and N,N-dicyclohexylcarbodiimide (DCC) in tetrahydrofuran, followed by purification of activated esters on silica column and then reaction with two-fold molar excess of c(RGDfK) in dimethylformamide (DMF) in the presence of triethylamine under argon atmosphere overnight (yield about 85%). Aminolysis of Bpheid-c(RGDfK) and Bpheid dissolved in chloroform-methanol mixture was induced with bis(3-aminopropyl)amine (yield >90%). The resulting Bchl-Ds were first purified using n-butanol-water extraction, and then by High-performance liquid chromatography (HPLC), using C_18_-column and acetonitrile-water gradient. HSA (90 mg) was activated with sulfoNHS and 1-Ethyl-3-(3-dimethylaminopropyl)carbodiimide hydrochloride (EDAC) (sulfoNHS:EDAC:HSA = 1000:500:1 molar ratio) in 50 mM phosphate buffer, pH 5.0. After 45 minutes, a sample of the reaction mixture (about 1 mL) was passed through PD-10 column (Sephadex G-25 M, GE Healthcare, Uppsala, Sweden) using 5 mL of the same buffer to recover the protein fraction (HSA-sNHS). Bchl-Ds (about 20 mg each) were dissolved in DMF (2 mL) and added to 2 mL HSA-sNHS, diluted with 4 mL of 50 mM phosphate buffer, pH 8.0, and 1 ml of 5% mannitol. Reaction mixtures were sonicated and stirred overnight under argon atmosphere, then evaporated to remove DMF, re-dissolved in water and passed again through PD-10 column for collection of Bchl-D-HSA-conjugates. To remove non-covalently bound Bchl-D, the products were evaporated and extracted several times with methanol. The extent of HSA conjugation was calculated from the molar ratios between the Bchl-D and HSA parts, determined spectrophotometrically and by Bradford assay, respectively. Approximately 10% of HSA was conjugated. Aliquots of conjugates equal to 0.7 nmol Bchl-D, were prepared for injections.

### Cell culture

MDA-MB-231 breast cancer cells were obtained from the American Type Culture Collection (Manassas, VA, USA). MDA-MB-231-RFP were generated by stable transfection of the cell line with pDsRed-Monomer-Hyg-C1 (Clontech, Palo Alto, CA, USA), which carries the hygromycin resistance gene in which the DsRed-Monomer gene was replaced with pDsRed2 (from pDsRed2-N1 plasmid, Clontech, Palo Alto, CA, USA). Lipofectamine™ 2000 (Invitrogen, Carlsbad, California, USA) was used for the transfection process according to the manufacturer's protocol. Stably RFP-expressing cells were then cloned and maintained in RPMI 1640 medium supplemented with 1 mmol/L sodium pyruvate, 10% fetal calf serum, 250 μg/ml hygromycin, 0.06 mg/ml penicillin and 0.1 mg/ml streptomycin.

### Association constants of Bchl-Ds to HSA

Association constants of Bchl-Ds to HSA were deduced from spectroscopic measurements of the ratio between Bchl-Ds and HSA for different concentrations of HSA using both factor analysis, as previously described [38] and by monitoring changes in the near infrared (NIR) absorption of the Bchl-Ds during titration with HSA. Briefly, all four Bchl-Ds present broad NIR absorption with reduced intensity in SA-free aqueous solutions. Upon addition of SA, they form 1:1 complexes with the added protein ((Bchl-D)/SA) with strong and narrow NIR absorption bands. The narrow-band absorption is proportional to the ((Bchl-D)/SA) concentration enabling calculation of the association constant *Ka *through the following equation:

Where [SA]_0 _is the analytical concentration of the added SA and [(Bchl-D/SA)] is the concentration of the Bchl-D complex with SA calculated from the spectral changes.

### Animals

Female, CD-1, nude mice (7 to 8 week old, about 25 g) were housed and handled with *ad libitum *access to food and water at the Core Animal Facility according to Institutional Animal Care and Use Guidelines. All experimental procedures were approved by the Institutional Animal Care and Use Committee at the Weizmann Institute of Science (Rehovot, Israel).

### Mouse tumor model

Mammary fat pads (left, bottom nipple) of female mice were inoculated with harvested MDA-MB-231-RFP human breast cancer cells (4 × 10^6 ^in 100 μl saline). Tumors were classified as 'small' (≤100 mm^3^) at one to two weeks from time of cell injection, or 'large' after three to four weeks (necrotic tumors, 250 to 500 mm^3^). To avoid tumor burden, mice were sacrificed (cervical dislocation) when tumor size reached 10% of body weight or at 90 days post-implantation. External caliper measurements, length (L), width (W) and depth (D) were used to calculate *in vivo *tumor volume according to the formula: V = L/2 · W/2 · D/2 · π · 4/3 [46].

### Whole-body fluorescence imaging

The *in Vivo *Optical Imaging System (IVIS^®^100/XFO-12, Xenogen Corp., Alameda, CA, USA) was used to acquire fluorescent images of the RFP-expressing tumors, as well as Bchl-D compounds associated with tissues and organs after their intravenous (i.v.) infusion to the tested animals. The field of view was 15 cm. RFP fluorescence, in units of photons/sec, was detected using 525/50 nm and 612/75 nm filter sets for excitation and emission, respectively, with one second integration time. NIR fluorescence of the different Bchl-Ds, in units of photons/sec, was detected using 680/30 nm and 847/75 nm filter sets for excitation and emission, respectively, at an integration time of five seconds. Background fluorescence for all quantitative analyses was calculated by recording the average fluorescence (photons/sec)/cm^2 ^from three animals in three different areas around and within the tumor of untreated animals provided with a similar diet for three days before measurements.

Drug accumulation was determined following i.v. administration of molar equivalents of the Bchl-D moiety to the tail vein of mice fed with a chlorophyll-free, purified diet (Harlan Teklad, Harlan Laboratories, Indianapolis, IN, USA) for three days before administration (in order to reduce skin and food autofluorescence). Before imaging, mice were anesthetized by intraperitoneum injection of a 30 μl mixture of 85:15 ketamine:xylazine.

Xenogen Living Image Software (Xenogen Corp., Alameda, CA, USA) was used for sequential fluorescent image acquisition [see Additional file 1] and superimposition of photographic images of mice and color-coded fluorescent images. Statistical analysis was performed using Origin8.1 (OriginLab, Northampton, Massachusetts, USA) and SPSS15 (SPSS Inc, Chicago, Illinois, USA) softwares.

### Biodistribution studies

Anaesthetized mice were i.v. injected with 15 mg/kg STL-6014 (n = 27) or 9 mg/kg STL-7012 (n = 15). At each indicated time point, three animals were sacrificed and blood, kidney, liver and tumor tissues were collected into pre-weighed vials and frozen at -20°C. Tissue samples were homogenized in methanol (100 mg tissue/ml) and extracted the next morning by centrifugation (13,000 × g five minutes) at room temperature. Supernatants were diluted in methanol by a factor of two and drug concentration was determined by fluorescence measurements (Varian - Cary Eclipse spectrofluorimeter, Palo Alto, California, USA) at 750 nm (peak values). Drug concentrations were interpolated from a fluorescence calibration curve, based on predetermined drug concentrations using absorption spectroscopy.

### *In vivo *competition between STL-6014 and free c(RGDfK) in MDA-MB-231-RFP tumors

STL-6014 (140 nmol, 7.5 mg/kg) was i.v. injected to the tail vein of mice one hour after being pre-injected with an excess (8.5 μmol) of c(RGDfK) free peptide. Whole-body fluorescence images were recorded up to 24 hours post-STL-6014 injection (IVIS^®^, Xenogen Corp., Alameda, CA, USA) with the respective filter settings, described above.

### Histology and *in vitro *calculations of tumor and necrosis volumes

Tumors were surgically removed from sacrificed mice, fixed in 3.7% formaldehyde, and embedded in paraffin blocks. Tumors were serially dissected to 4 μm slices with a separation of 0.5 mm between consecutive slices. Slices were stained with H&E under standard conditions and evaluated for pathological markers (E800 microscope equipped with a Nikon DXM1200 digital camera, magnification × 0.6, objective × 0.5, Nikon, Tokyo, Japan).

Tumor volume (Tv), necrosis volume (Nv), the total tumor area (Ta) and necrotic area (Na) of each slice were calculated using Image Pro Plus 0.5 software. Tv and Nv were calculated using the following equations:

where n is the number of slices for the evaluated tumor.

## Results

### Necrotic MDA-MB-231-RFP tumor model

**Figure 2:**
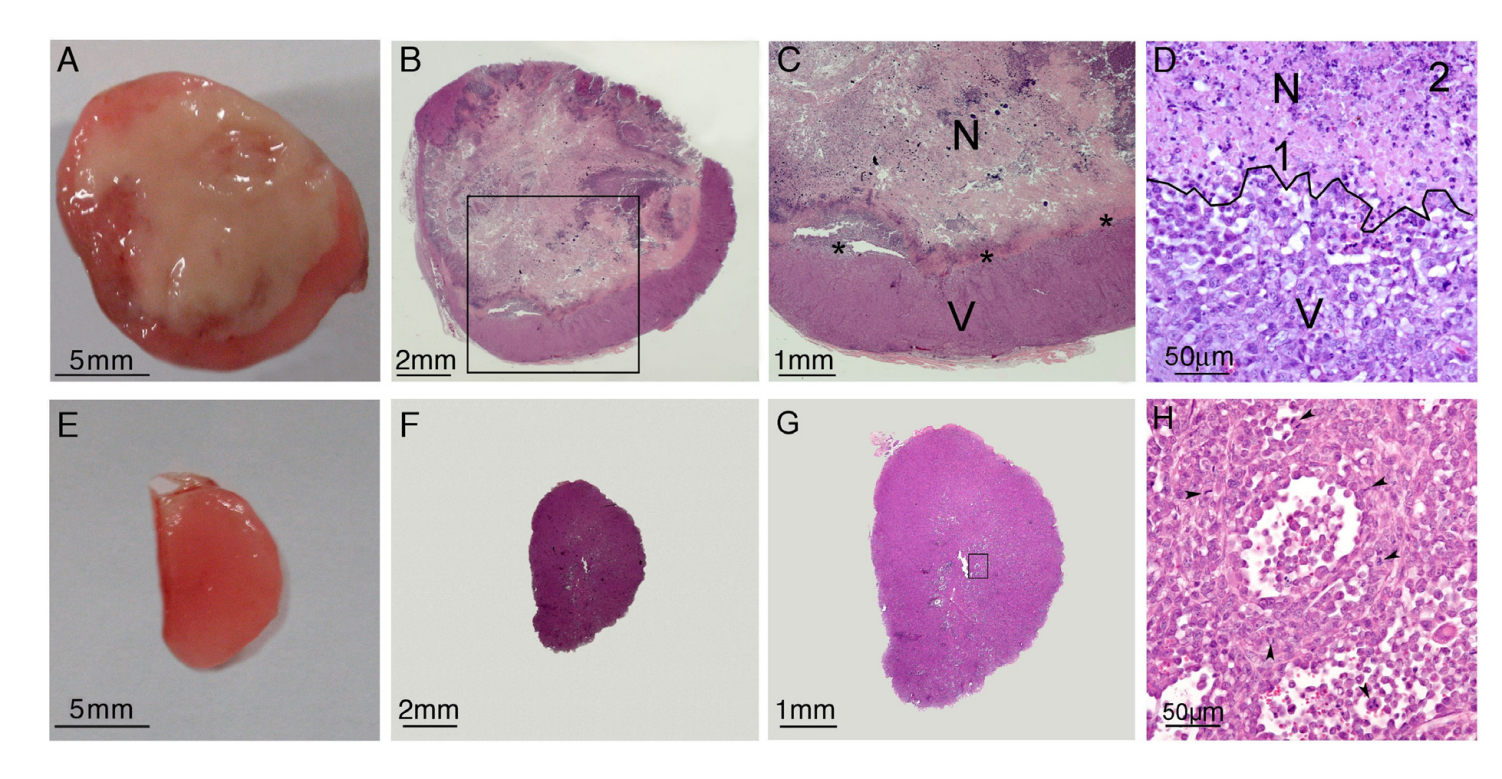
**Macroscopic and microscopic features of large and small MDA-MB-231-RFP tumors**. Small (about 100 mm^3^) and large (250 to 500 mm^3^) orthotopically-grafted MDA-MB-231-RFP tumors were excised at pre-defined time intervals from cell inoculation. **(a) **Macroscopic appearance of a cross section of a freshly excised large tumor. The bulk of the mass is composed of opaque, necrotic tissue, while the viable tissue is limited to the rim. **(b) **Subgross appearance of the tumor shown in (a). There is very good correlation between the macroscopic and microscopic features. The necrotic tissue in the center is partly surrounded by a rim of viable tissue. **(c) **Medium magnification of the boxed area in (b). The interface between necrotic (N) and viable (V) tissue is indicated with asterisks. **(d) **High magnification of a region at the interface between necrotic (N) and viable (V) tissues. The border between the two zones is marked by a black line. Areas with predominant karyolysis (1, pink tissue) and predominant karyorrhexis (2, granular basophilic nuclear debris) are identified. **(e) **Macroscopic appearance of a cross section of a freshly excised small tumor. The tissue is homogenous and there is no evidence of necrosis. **(f) **Subgross appearance of the small tumor shown in (e) at the same magnification as (b). **(g) **At slightly higher magnification, the homogenous appearance of the tumor is evident. **(h) **Boxed area - neoplastic viable cells; Arrowheads - mitotic structures.

Small (≤100 mm^3^) and large (250 to 500 mm^3^) MDA-MB-231-RFP tumors were excised at pre-defined time intervals after cell inoculation for morphological analysis. Large tumors (Figures 2a to 2d) featured an extensive central eosinophilic and often hypereosinophilic (Figures 2b and 2c) necrotic domain with widespread karyolysis and minimal karyorrhexis and pyknosis (Figure 2d). In addition, mild, multifocal, neutrophilic infiltration was seen at the margins of the necrotic tissue. Viable areas were limited to the periphery of the tumor and included disorganized proliferation of neoplastic cells arranged into dense cellular sheets. The neoplastic cells were round to irregular with a high nuclear/cytoplasmic ratio and irregular vesicular nuclei (Figure 2d). Figures 2e to 2h are representatives of small tumors, and illustrate homogenous tissue with no evidence of necrosis.

Above 90% of the large tumors (n = 30, 250 mm^3 ^≤Tv ≤500 mm^3^), presented central necrosis that occupies about 50% of Tv. Five to ten percent of the large tumors (n = 2) presented smaller volume of necrosis (about 50 mm^3^, about 15% of Tv). Conversely, more than 90% of the small tumors (n = 20, Tv ≤ 100 mm^3^) presented almost no necrosis. In 10% or less of these tumors (n = 2) a central necrosis (Tv ≤ 15 mm^3 ^≤20% of Tv) is observed. Thus, central necrosis appears to correlate only with large tumors of MDA-MB-231-RFP cells in this mouse model.

### STL-6014 uptake into orthotopic MDA-MB-231-RFP tumors

The tumor-targeting capabilities of RGD-conjugated Bchl-Ds, was tested by following the accumulation of i.v. injected STL-6014 in mice bearing large or small MDA-MB-231-RFP tumors.

#### Large tumors

**Figure 3:**
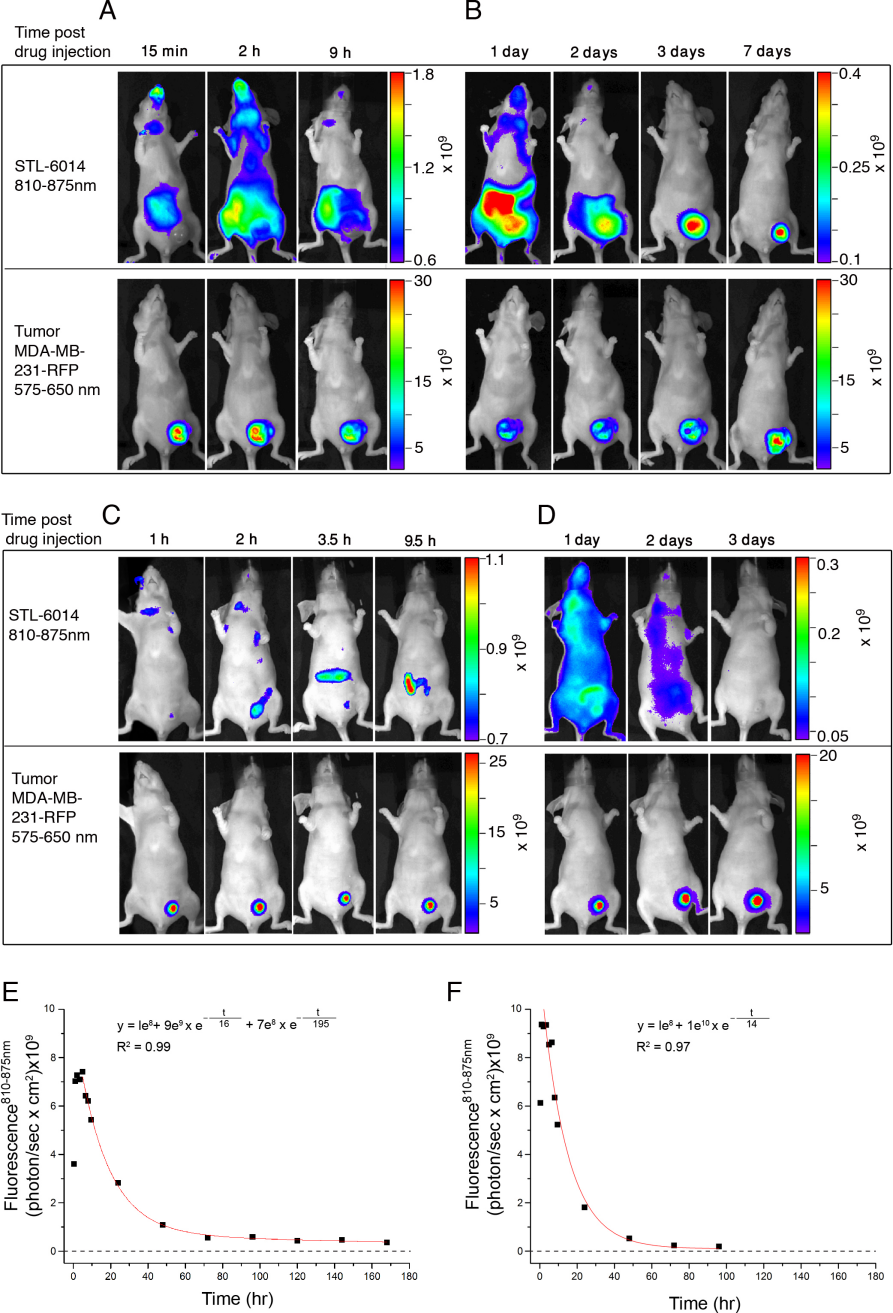
**Accumulation of STL-6014 in large and small MDA-MB-231-RFP tumors**. CD-1 nude, female, mice bearing orthotopically-grafted MDA-MB-231-RFP tumors were intravenously injected with STL-6014 (15 mg/kg). Fluorescent images of **(a and b) **large (n = 9) and **(c and d) **small (n = 10) tumors, were taken at the indicated times post-injection. Upper panels - near infrared (NIR) fluorescence images indicate STL-6014 distribution, lower panels - red fluorescence images indicate tumor size and location. **(a) **Fluorescence signals in a large tumor over the first nine hours post-administration. **(b) **Fluorescence signals in the large tumor over the following one to seven days post-administration. **(c) **Fluorescence signals in a small tumor over the first 9.5 hours post-administration. **(d) **Fluorescence signals in the small tumor over additional one to three days post-administration. Longitudinal STL-6014 decay curves in representative **(e) **large and **(f) **small tumors. Total fluorescence intensity within the individual tumor boundaries, was normalized per unit area and expressed as photon/(sec × cm^2^). Other details are as indicated in the methods section.

Dynamic, whole-body fluorescence imaging was first performed at specific times for up to seven days after drug injection to mice with large tumors (n = 9, Figures 3a and 3b, upper panels). Nine days follow-up is further provided in additional file 2. The location and sizes of the respective tumors were verified by whole-body RFP fluorescence scans (Figures 3a and 3b, lower panels). Extensive fluorescence was detected throughout the animal body within the first 15 minutes after STL-6014 injection (Figure 3a, upper panel), reflecting high levels of circulating drug in the blood and clearance organs (i.e. liver, kidney). On day one post-administration, STL-6014 had markedly accumulated in the tumor, relative to the surrounding tissue, with considerably lower levels in the abdominal clearance organs. On day three, the clearance organs appeared to be almost STL-6014-free, while the fluorescence in the tumor was retained (Figure 3b, upper panel). The fluorescence intensity in the individual clearing organs at the relevant time points [see Additional file 3], correlate well with the regional fluorescence decline in the whole body images.

#### Small tumors

Accumulation of STL-6014 in small tumors (n = 10) was assessed under the same experimental conditions but appeared markedly different (Figures 3c and 3d). Although peak fluorescence of the injected drug was evident within the tumor at six to eight hours after injection (Figure 3c, upper panel), its level relative to the surrounding tissue was rather small. Tumor fluorescence returned to background values within 48 h of drug injection (Figures 3c and 3d).

### Quantitative assessment of the STL-6014 dynamic fluorescence in small and large tumors post i.v. administration

The temporal evolution and decay of STL-6014 fluorescence density ((number of photons at (810 to 870 nm))/(sec × cm^2 ^tumor tissue)) was calculated and presented versus time for mice bearing large tumors (n = 9, Figure 3E) or small tumors (n = 10, Figure 3f). In both tumor types, STL-6014 concentrations peaked within six to eight hours of administration. This period of accumulation was then followed by a mono-exponential decay in the small tumors, with an average t_1/2 _value of 14.0 ± 1.5 (SD) hours (R^2 ^≥ 0.97, *P *< 0.001, n = 10). In contrast, clearance from the large, necrotic tumors exhibited a bi-exponential decay with average t^1^_1/2 _values of 12.0 ± 3.5 hours during the first 24 hours and t^2^_1/2 _of 210.0 ± 86.0 hours thereafter (R^2 ^≥ 0.98, *P *< 0.001, n = 9). No statistically significant difference was seen between the fluorescence decay constant in small tumors and the first decay constant of large tumors. Large tumors maintained significant concentrations of STL-6014 for prolonged periods, as evident by the tumor fluorescence still detectable at nine days, and similar to the levels detected on day seven post-administration [see Additional file 2]. Thus, the emerging situation is of a relatively rapid-in slow-out pattern of accumulation. These results were substantiated by detailed analysis of drug concentrations in the different organs/tissues, as described in the 'biodistribution and pharmacokinetics analysis' section below.

Notably, the RFP fluorescence from the tumor cells declined shortly after STL-6014 infusion (Figures 3a to 3d lower panels) and then increased again at rates that appeared to mirror the uptake (first hours) and clearance of the STL-6014 from the corresponding tumors. Hence, this phenomenon provides independent evidence for the differential accumulation and clearance patterns of STL-6014 in small and large tumors [see Additional file 4].

### Selective STL-6014 accumulation in the necrotic domain of MDA-MB-231-RFP tumors

**Figure 4:**
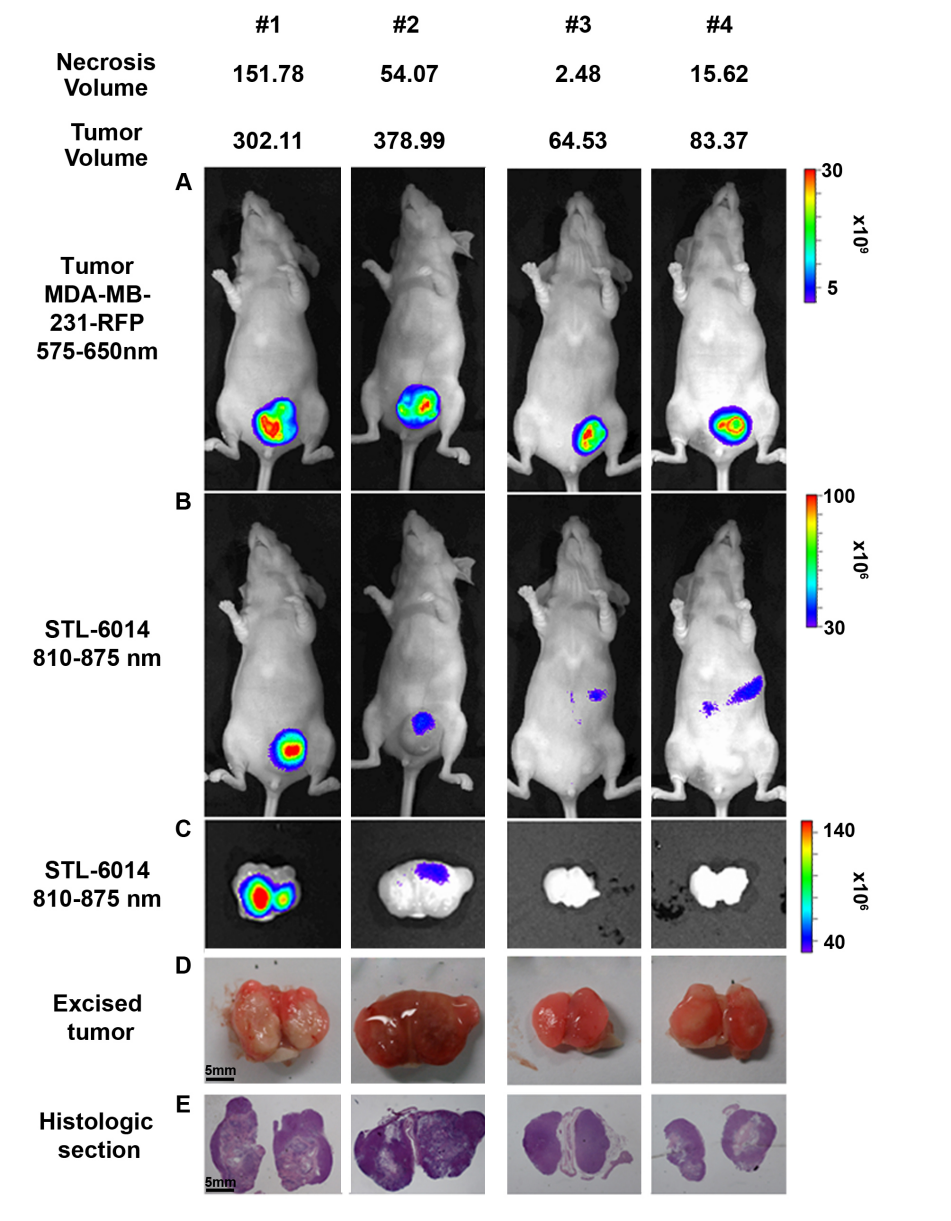
**Correlation between necrotic volumes and STL-6014 accumulation in MDA-MB-231-RFP tumors**. CD-1 nude, female mice bearing MDA-MB-231-RFP tumors orthotopically grafted to the mammary fat pad were intravenously injected with STL-6014 (15 mg/kg). Images of large (animals #1,2) and small (animals #3,4) tumors were taken at seven and five days, respectively, post-injection. **(a) **Red fluorescence protein (RFP) fluorescence images indicate tumor size and location. **(b and c) **Near infrared (NIR) fluorescence images demonstrating STL-6014 distribution both *in vivo *and after dissection, respectively. **(d) **Color photos. **(e) **Tissue sections of the tumor center stained with hematoxylin and eosin.

Figure 3 shows that the fluorescence of STL-6014 accumulates and then clears very slowly from large MDA-MB-231-RFP tumors whereas clearance from small tumors is more than 10-fold faster (see above). As more than 90% of the large tumors have large necrotic volumes and more than 90% of the small ones are non-necrotic, we propose that t^1^_1/2 _and t^2^_1/2 _monitor clearance of STL-6014 from the viable and necrotic tumor domains, respectively. An alternative possibility is that the slow fluorescence decay in the large tumors reflects diffusion barriers that are related with the tumor volume regardless of the necrosis. Hence, we next examined the relation between prolonged accumulation of STL-6014 within the tumors (on days five and seven post administration for small and large tumors, respectively) and tumor necrosis that was independently assessed by tumor dissection and histopathology as described above, following the whole body dynamic assay. We thus obtained four tumor classes: (1) typical large tumors (Tv ≥ 250 mm^3^, Nv ≥ 150 mm^3^); (2) non-typical large tumors (Tv ≥ 250 mm^3^, Nv about 50 mm^3^); (3) typical small tumors (Tv ≤ 100 mm^3^, Nv ≤ 6 mm^3^), and (4) non-typical small tumors (Tv ≤ 100 mm^3^, Nv about 10 to 20 mm^3^). The left panel in Figure 4 illustrates representative histological sections and STL-6014 fluorescence images from the whole body and dissected tumors of groups number 1 and number 2, and the right panel of groups number 3 and number 4. These images clearly show that the STL-6014 fluorescence only sustains prolonged high intensity in tumors with large Nv values. This conclusion is substantiated by the observations that animal number 1 in Figure 4 left panel (Nv = 151 mm^3^) presents significantly higher fluorescence than animal number 2 (Nv = 54 mm^3^) although their Tv values are similar. Thus, as more than 90% of the large tumors are necrotic and more than 90% of the small tumors are non-necrotic, this experiment shows that prolonged fluorescence of STL-6014 at more than three days post injection is associated with large Nv and not Tv.

**Figure 5:**
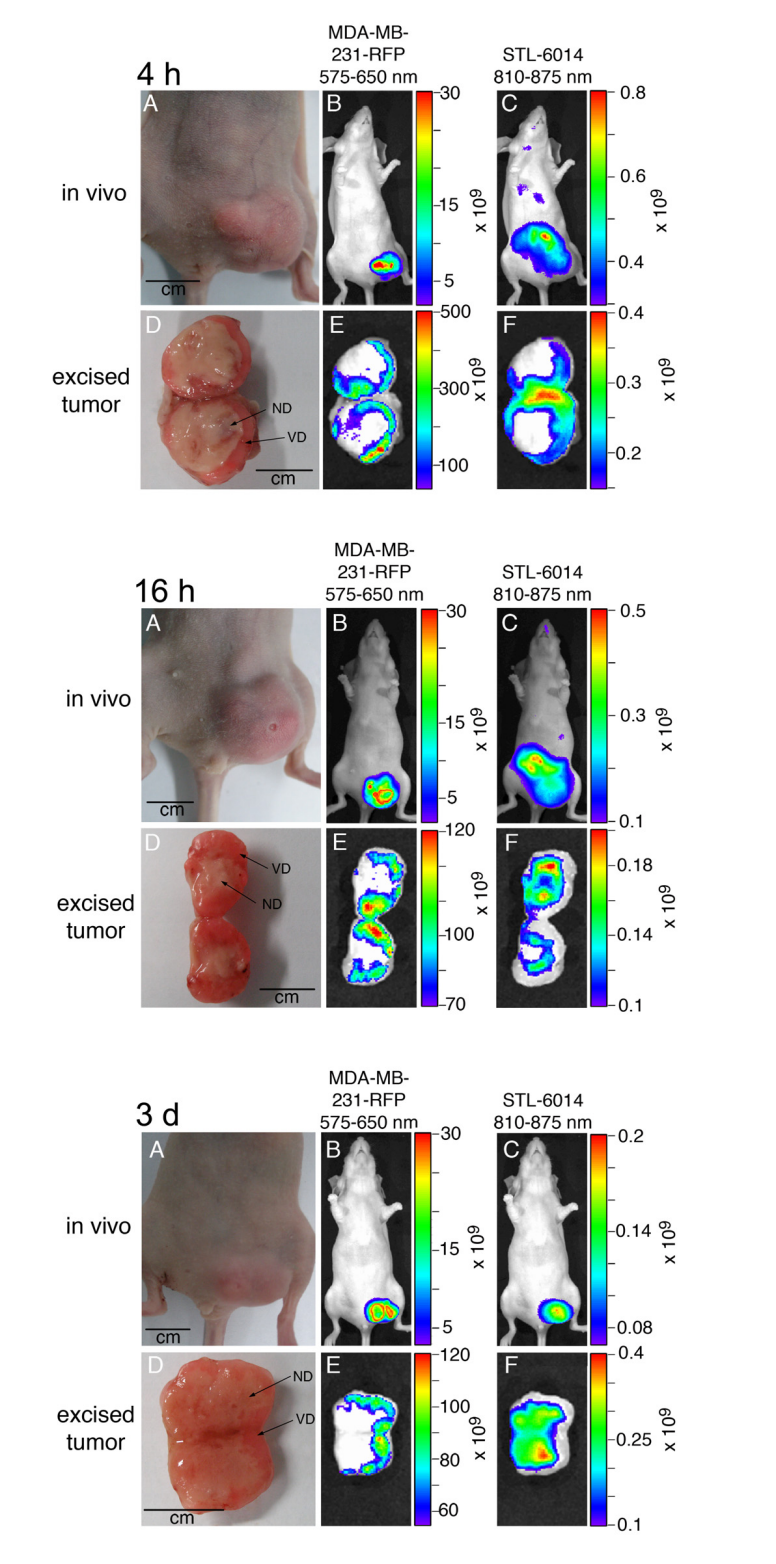
**STL-6014 accumulation in the necrotic area of large orthotopic MDA-MB-231-RFP tumors**. CD-1 nude, female mice (n = 3) bearing an orthotopically grafted, large MDA-MB-231-RFP tumor were intravenously injected with 15 mg/kg STL-6014. Whole-body and excised tumor images were taken at 4 hours, 16 hours, and 3 days post-injection. Upper panels - *in vivo *images, lower panels - images of excised tumors. **(a and d) **Color photos. **(b and e) **Red fluorescence images for detection of tumor location and size. **(c anf f) **near infrared (NIR) fluorescence images for detection of STL-6014 distribution. All other details are as described in the methods section. ND, necrotic domain, VD, viable domain.

Next, the spatial distribution of the STL-6014 was monitored in dissected typical large tumors by following its fluorescence at eight time points (10 minutes, 1, 4, 16, and 24 hours and 3, 5, and 7 days, n = 3 for each time point). Images for representative time points are shown in Figure 5 and additional file 5. At four hours post injection, STL-6014 signal was observed within the viable tumor region alone. At 16 hours post-injection, a distinct signal intensification was observed towards the center of the necrotic domain. Beyond that time point, fluorescence was only observed in the necrotic domain. Importantly, the clear demarcation between the RFP fluorescence (originating in the tumor viable domain) and the STL-6014 fluorescence (from the necrotic domain) did not shift towards the tumor center during these days of examination (Figure 5) [see also Additional file 5].

Cumulatively, these results illustrate progressive accumulation and selective retention of STL-6014 within the necrotic tumor domain after temporary lodging in the tumor viable region.

### Biodistribution and pharmacokinetics of STL-6014

**Figure 6:**
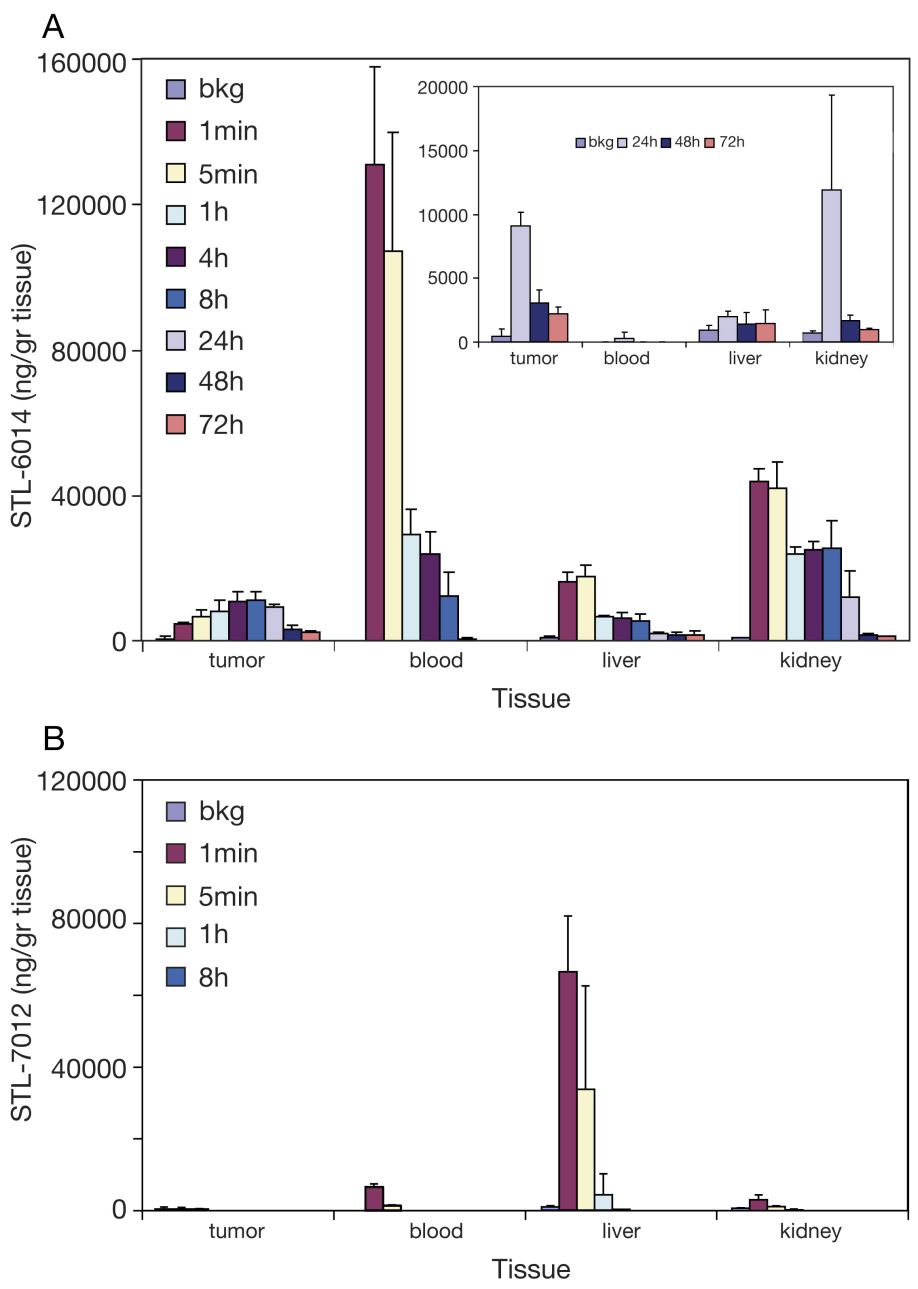
**Biodistribution of STL-6014 and STL-7012 in MDA-MB-231-RFP tumor-bearing mice**. CD-1 nude, female mice (n = 3 for each time point) were intravenously injected with: **(a) **15 mg/kg STL-6014 or, **(b) **9 mg/kg STL-7012 and sacrificed at the indicated times. Tissue samples were collected for STL-6014 or STL-7012 extraction. Values represent averaged fluorescence intensities (± standard deviation), measured as described in the methods section.

STL-6014 levels in blood, liver, kidney and large MDA-MB-231-RFP tumors were assessed at the indicated time points (Figure 6a). STL-6014 reached peak concentrations in the tumors at eight hours post-injection (11 μg drug/gr tumor tissue; about 3.0% of the initial drug dose), while its levels in the normal tissues examined peaked at less than five minutes post-injection and cleared to nearly background levels within less than 72 hours Tumor STL-6014 levels were about two orders of magnitude higher than in the blood during the 48 to 72 hours post-administration period (Figure 6a, insert), about 10-fold higher than in the spleen, heart, brain, fat and muscle (data not shown), and approximately two-fold higher than in the kidney, liver (Figure 6a, insert), intestines, lung and skin (data not shown). STL-6014 underwent rapid hepatic clearance with a t_1/2 _of about four hours. Because of the relatively fast clearance of STL-6014 from the non-necrotic tumors, the fluorescence difference between the tumor and the surrounding normal tissue sustained for short time accounting for the poor imaging of these tumors as presented in Figures 3c and 3d. These results substantiate our hypothesis that STL-6014 and similar agents can be used for selective imaging of necrotic tumors.

### Is the c(RGDfK) moiety essential for the STL-6014 uptake by the tumor?

**Figure 7:**
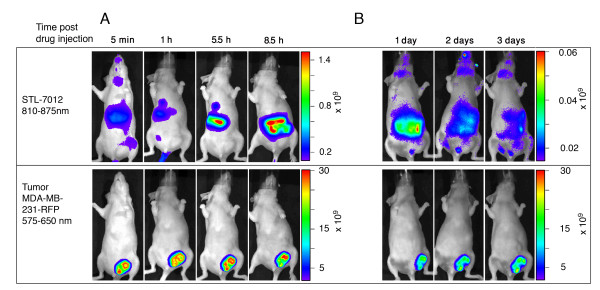
**The role of the c(RGDfK) moiety in driving Bchl-D accumulation within tumors**. CD-1 nude, female mice (n = 3) bearing an orthotopically grafted, large MDA-MB-231-RFP tumors were intravenously injected with 9 mg/kg STL-7012. Drug accumulation was monitored, by means of fluorescence imaging, for up to three days and a representative animal is shown. Upper panel - near infrared (NIR) fluorescence images indicate bacteriochlorophyll derivative (Bchl-D) distribution and lower panel - Red fluorescence images indicate tumor location.

In order to examine if the RGD moiety is imperative for the selective uptake of STL-6014 by the tumor, we compared its time-dependent accumulation within tumors, with that of the RGD-free STL-7012 (Figure 1). Quantitative assessment of the STL-7012 fluorescence intensity from tissues extracts was performed (Figure 6b) showing no STL-7012 accumulation in the tumor at any time longer than one hour post administration. No specific tumor fluorescence of STL-7012 was seen in mice bearing large, necrotic tumors, already at one hour post i.v. injection (Figure 7, n = 3). Similar results were obtained in mice bearing small tumors (data not shown). These findings clearly indicate that the c(RGDfK) moiety is imperative to Bchl-D accumulation and retention in both small and large tumors.

### The role played by the Bchl-Ds association to SA in their uptake and prolonged accumulation in the MDA-MB-231-RFP tumors

**Figure 8:**
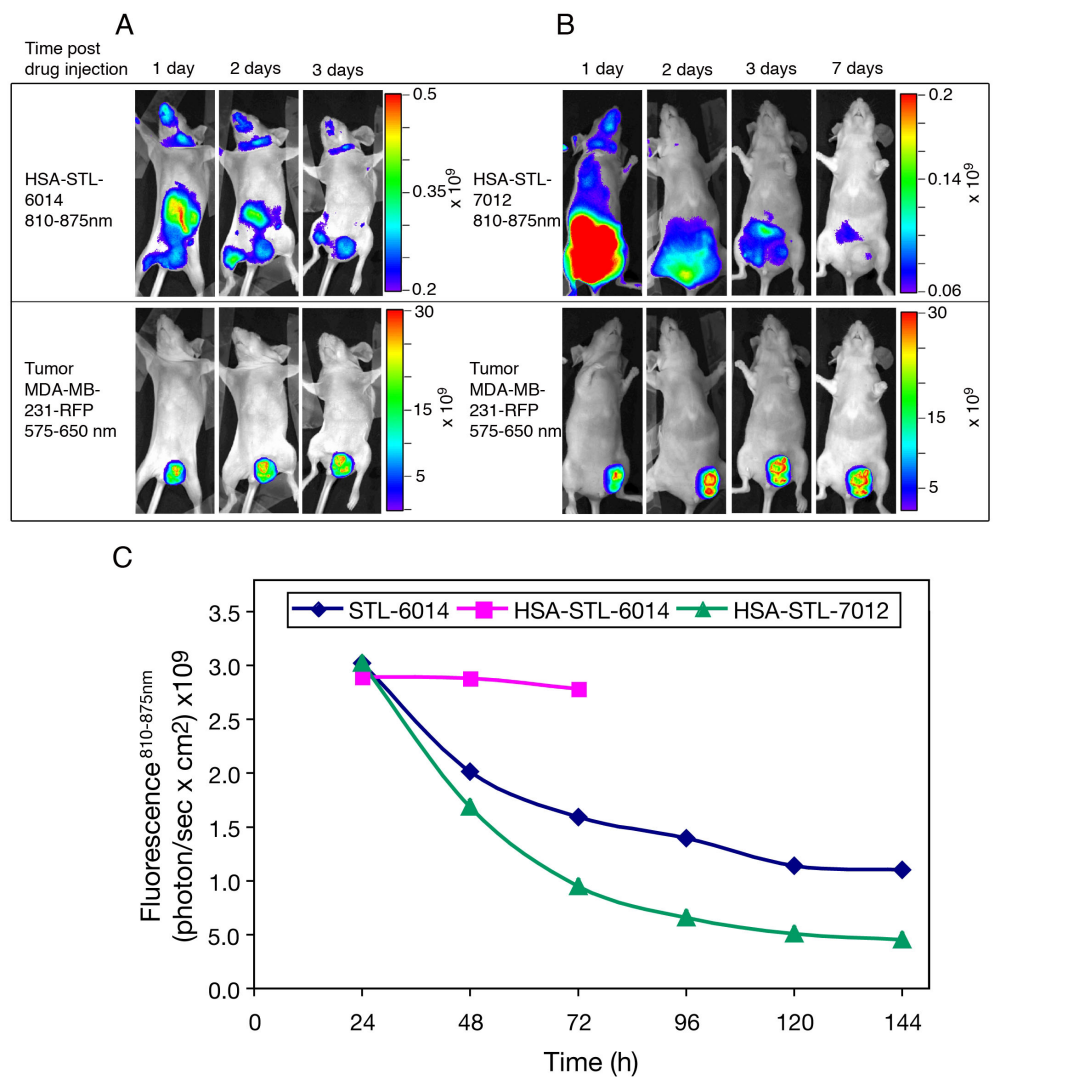
**Accumulation of HSA-STL-6014 and HSA-STL-7012 in large MDA-MB-231-RFP tumors**. CD-1 nude, female, mice bearing orthotopically grafted, large MDA-MB-231-RFP tumors were intravenously injected with 0.7 nmol **(a) **HSA-STL-6014 or **(b) **HSA-STL-7012. Fluorescence images of the tumors were taken at the indicated times post-injection. Upper panel - near infrared (NIR) fluorescence images indicate bacteriochlorophyll derivative (Bchl-D) distribution, lower panel - red fluorescence images indicate tumor size and location. **(c) **Longitudinal accumulation of STL-6014, HSA-STL-6014 and HSA-STL-7012 in large tumors. Total fluorescence intensity within the individual tumor boundaries at the indicated times, was normalized per unit area and expressed as photon/(sec × cm^2^).

In our previous studies [28,38] we have demonstrated that the water soluble WST11 is primarily carried in the circulation through non-covalent associations with SA. However, such non-covalent association appears insufficient to drive tumor accumulation and retention of WST11 [44] and STL-7012 (Figures 6b and 7). On the other hand when STL-7012 was covalently bound to HSA, it presented some accumulation and prolonged retention in the tumor (Figures 8b and 8c) and HSA-STL-6014, appears to clear extremely slowly, if at all, from the tumor (Figures 8a and 8c). These findings corroborate with the aforementioned finding that covalent binding or non-covalent association of contrast agents with SA, significantly enhances their uptake by tumors [26].

### Does the Bchl-D conjugation to the c(RGDfK) increases its binding affinity to SA and thereby tumor accumulation and retention?

Following the above findings, it may be suggested that stronger binding of STL-6014 to SA compared with RGD-free STL-7012 analogue, accounts for the increased accumulation of the former in tumors. Therefore, we set out to determine the association constants of the four Bchl-Ds presented in Figure 1 to HSA. The derived constants (Figure 1, in parenthesis) were found to be within the same order of magnitude. In fact, those for the RGD conjugates were four to six fold lower than the association constants of their RGD-free analogues, ruling out stronger SA association as the sole basis for STL-6014 retention in the tumor.

In summary, association to SA appears to be important for the uptake of Bchl-Ds to the necrotic tumor domain, but is not enhanced by the RGD moiety.

### Does the contribution of the RGD moiety involve association with tumor-specific cell receptors?

As c(RGDfK) was found not to enhance the non-covalent association of the Bchl-Ds moiety (e.g. STL-7012) with circulating SA, emphasis was placed on defining the role of RGD in STL-6014 uptake by the tumor. The possible involvement of a specific RGD binding site such as αVβ3 integrin in the observed drug accumulation within tumors was next examined by designing a competition assay between STL-6014 and free c(RGDfK).

#### Small tumors

**Figure 9:**
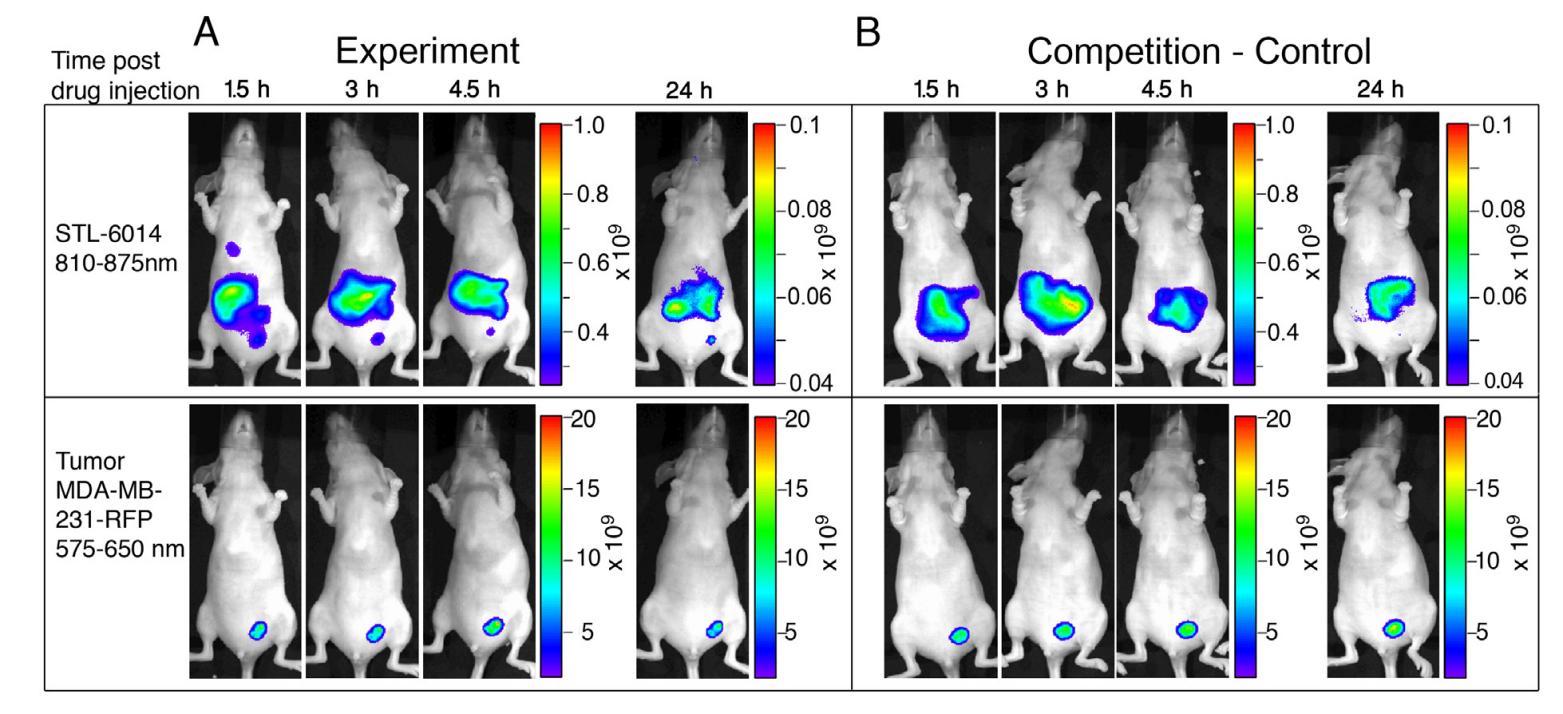
***In Vivo *inhibition of STL-6014 accumulation in small orthotopic human breast MDA-MB-231-RFP tumors by c(RGDfK)**. **(a) **CD-1 nude, female mice bearing orthotopically grafted MDA-MB-231-RFP (n = 3) were intravenously injected with 140 nmol STL-6014. **(b) **CD-1 nude, female mice bearing orthotopically grafted MDA-MB-231-RFP (n = 3) were intravenously injected with 8.5 μmol c(RGDfK) and one hour later with 140 nmol STL-6014. Fluorescent images were taken at the indicated times post-injection. Upper panel - near infrared (NIR) fluorescence images indicate bacteriochlorophyll derivative (Bchl-D) distribution, lower panel - red fluorescence images indicate tumor size and location. All other details are as described in the methods section.

STL-6014 (7.5 mg/kg, 140 nmols/mouse) was i.v. injected to mice bearing small tumors one hour after the injection of a 61-fold molar excess of free c(RGDfK) (Figure 9). When delivered alone, STL-6014 fluorescence was retained within the tumor throughout the experiment (Figure 9a). In contrast, in the presence of free c(RGDfK), competing for the same binding sites (Figure 9b), almost no STL-6014 fluorescence was detected in the tumor. Thus, the RGD moiety plays a critical role in drug uptake to small tumors by interacting with specific receptors in the tumor tissue.

#### Large tumors

A similar competition experiment was performed for animals with large tumors. However, in this case the uptake and accumulation of STL-6014 was not affected by the pre-administration of free c(RGDfK) (data not shown).

## Discussion

Preferential targeting of contrast and therapeutic agents for detection, imaging and treatment of tumors is understood to bear great prospects for effective cancer management. Of particular significance is the early, *in situ *detection of necrotic tumors in patients with localized breast cancer. This is critical for the appropriate selection of a treatment approach.

This study introduces a new strategy for *in vivo *imaging of necrotic breast tumors, following the design of Bchl-D-based agents that associate non-covalently with circulating SA immediately upon i.v. administration, and contain ligands with acute affinity for tumor-specific receptors. The moderate association of Bchl-D with SA was postulated to assist extravasation and temporal retention of the Bchl-RGD molecules by the necrotic tumor domains, via the EPR effect, while their strong affinity to tumor-specific receptors would maintain prolonged residence in these domains.

To test this hypothesis, we introduced the STL-6014 conjugate (Fig. 1) to mammary tumor-bearing mice. Although STL-6014 moderately associates with SA (Ka = 1.2 × 10^5^M^-1^, Figure 1), the c(RGDfK) moiety has been shown to strongly associate (K_a _about 10^9^M^-1^) with αVβ3 integrin receptors abundant in the tumor vasculature, the cell membrane of some cancer cells [42] and on macrophages that infiltrate to the tumor tissue. Hence, c(RGDfK) was expected to provide high tumor affinity to the tested RGD-Bchl-D conjugates during their circulation as complexes with SA.

By correlating the red fluorescence of the RFP-expressing cancer cells and the NIR fluorescence emitted by the different Bchl-Ds, we monitored the dynamics of their biodistribution, as well as their accumulation in the tumor, prolonged retention and compartmentalization in the tumor sub-domains. The presented data demonstrate that conjugation to c(RGDfK) and complexation with SA synergistically enhances the tumor uptake and retention of the tested RGD-Bchl molecules in tumor necrotic domains. The c(RGDfK) moiety was shown to be essential for tumor-specific drug accumulation, as evidenced by the lack of free unconjugated Bchl-D uptake (Figure 7) [44]. On the other hand the half life of STL-6014 (Figure 3) was much longer even in small tumors (about 12 hours) than that of free c(RGDfK) molecules in a similar animal model (minutes) [43]. This role is further demonstrated by the significantly slower clearance of the HSA-STL-6014 covalent conjugate compared with the HSA/STL-6014 complex (Figure 8c) and the HSA-7012 covalent conjugates (Figures 8b and 8c) compared with HSA/7012 complexes (Figures 6b and 7). The same observations were recently made after i.v. administration of STL-6009, a non-fluorescent c(RGDfK)-(Pd)Bchl-D [44].

As the c(RGDfK) moiety was found not to increase the Bchl-Ds association to the SA, its key role presumably reflects the hypothesized affinity to particular receptors in the tumor tissue. Intense affinity to αVβ3 integrins receptors has been previously proposed to account for the c(RGDfK)-based drug retention-enhancing effect. MDA-MB-231 human breast cancer cells, as well as tumor vasculature have been shown to express the αVβ3 receptor [47,48]. In a recent study [44], we clearly correlated STL-6009 uptake by tumor xenografts of various cell types, with both their vascular density and the level of αVβ3 integrins expressed [44]. In the present study, the free circulating c(RGDfK) competition (Figure 9) provides additional evidence with regard to the role of RGD in active agent accumulation within tumors. However, the observation that administration of free c(RGDfK) molecules cannot compete with the uptake and accumulation of RGD-Bchl by large necrotic tumors is not in line with this simple explanation.

The differential response of large and small tumors to free c(RGDfK), complements the dramatic difference in STL-6014 accumulation patterns within the two tumor classes. Small tumors accumulated STL-6014 over the first four to eight hours post-drug injection, followed by its full clearance within less than two days. In contrast, large MDA-MB-231-RFP tumors containing necrotic regions accumulated STL-6014 at approximately the same rate, followed by a shift in STL-6014 localization to the central necrosis, where it reached peak concentration by 48 to 72 hours post-injection. This phase was followed by slow, bi-phasic clearance (Figures 3, 4 and 6a). The diverse clearance patterns were experimentally correlated with the viable and necrotic domains in the tumors, where the more rapid clearance was seen from viable regions. This conclusion was substantiated by findings that the fluorescence from 'large' tumors bearing small Nv, cleared significantly faster than from tumors of similar size but with larger Nv, namely smaller viable cells mass (Figure 4). The observation that the majority of 'small' tumors contained no or very small Nv (Figure 4) coincided with their monoexponential and fast fluorescence decay. Conversely, more than 90% of the large tumors featured large Nv, correlating with the bi-exponential clearance pattern (Figures 3, 4 and 6) [see Additional file 2].

In line with these observations, we propose the presence of two distinct Bchl-D-c(RGDfK) target sites. The first is suggested to comprise RGD-binding sites, presumably integrin receptors presented on neoendothelial cells in both small and large tumors. This site is further suggested to be easily accessible to both c(RGDfK)-Bchl-D and free c(RGDfK). The second target site correspond to the necrotic domains, involving integrin receptors presented by the regular tumor cells, eosinophylic necrotic tumor cells, and possibly infiltrating white blood cells. Interaction of RGD ligands with these integrins requires their extravasation and retention into the tumor interstitium. Such extravasation can be driven in the large necrotic tumors by the EPR effect that is relevant to the large c(RGDfK)-Bchl-D/SA complexes but not to the small, free c(RGDfK) molecules, which rapidly clear from the tumor [43]. Therefore, the free c(RGDfK) molecules cannot compete with c(RGDfK)-Bchl-D binding to cellular integrins in the large tumors. The prolonged retention of the c(RGDfK)-Bchl-D/SA within the large tumor shifts the equilibrium of the SA-bound Bchl-D-c(RGDfK) to a more stable, RGD-mediated interaction with the stationary integrin receptors through the RGD moiety. All together, the above findings indicate that the EPR effect may support mobilization of SA-associating therapeutic and imaging agents into the tumor necrotic domain. However, interactions with tumor-specific receptors are required for the prolonged retention and accumulation of these compounds within the tumor.

The sharp displacement of STL-6014 fluorescence from the viable to the necrotic domain within large tumors at increasing times after administration (Figures 4 and 5) [see also Additional file 5] presents a feature essential to diagnostic protocols in search of advanced tumors. This shift is believed to be driven by the diffusion pressure created by the imbalanced concentrations of c(RGDfK)-Bchl-D in the viable versus necrotic tumor domains. Neutrophils and possibly macrophages, which infiltrate the necrotic domain (Figure 2) [1] may participate in anchoring the migrating c(RGDfK)-Bchl-D molecules via αVβ3 integrin receptors, expressed at high levels [49,50]. Alternatively, self-aggregation of c(RGDfK)-Bchl-D molecules within the semi-aqueous necrotic domain may occur due to their increased concentrations, shifting the equilibrium toward further accumulation. These possibilities are currently under investigation. Notably, Chen and colleagues [26], recently showed that covalent binding of specific cyclic RGD molecules to HSA enhances their *in vivo *accumulation in tumors compared with the HSA-free analogues. They investigators related this phenomenon to the increased circulating t_1/2 _of the covalent conjugates (21 minutes vs. two to three minutes).

The reproducible results presented in this study have been further supported by analysis of STL-6009 and STL-6014 behavior in the same MDA-MB-231-RFP tumors and in human MLS ovarian and renal cell carcinoma tumors, respectively, at various stages of necrotic development (data not shown).

## Conclusions

Cumulatively, the presented data demonstrates selective accumulation and prolonged retention of c(RGDfK)-Bchl-D within the necrotic tumor domain in MDA-MB-231-RFP bearing mice. A clear reliance on the synergistic effect of both the RGD moiety and the agent's association to SA was illustrated.

Although the experiments described here utilized fluorescence for the selective imaging of necrotic tumors, they are highly relevant to the clinical setting. First, the orthotopic engraftment of human breast cancer to the mouse mammary pad offers an environment resembling human breast tumors. In addition, the central necrosis described here, reflects the natural development of breast tumors in humans. Furthermore, the RGD-mediated accumulation characterized in the present study is of relevance to human cancerous environments, where αVβ3 integrin expression is typically associated with neovessels, tumor cells and macrophages abundant in the necrotic tumor domain [47].

The described RGD/SA-mediated homing and prolonged retention of fluorescent Bchl-Ds in the tumor necrotic domain may enable early detection of tumor growth and foster prognosis and the development of novel modes of treatment, including drug-targeted photodynamic therapy. As previously reported, RGD-SA contrast agents [26] are likely to be immunogenic and/or toxic in the clinical setting. The strategy suggested in the present manuscript is not only simpler, but more versatile and expected to present minimal toxicity. The relatively rapid clearance of STL-6014 and other RGD-Bchl-Ds from the liver and spleen is of utmost importance in clinical application of developed drugs. Clearly, the use of STL-6014 in the clinical setting is limited by the optical technologies required for in-depth 3 D tumor detection. However, optical tomography of the breast can be useful for this purpose. Moreover, ^64^Cu and Mn Bchl-RGD conjugates have been recently prepared in our laboratory [37] and using inductively coupled plasma mass spectroscopy showed to undergo similar patterns of accumulation and retention in tumors as STL-6014. These new drugs are expected to open the way for *in vivo *imaging of deeper necrotic tumors, both in the preclinical and clinical settings using PET scan and MRI spectroscopies, respectively.

## Abbreviations

Bchl-D: bacteriochlorophyll derivative; Bpheid: bacteriopheophorbide *a*; c(RGDfK): cyclic arginine-glycine-aspartic acid-(D)phenylalanine-lysine; DCC: N,N-dicyclohexylcarbodiimide; DCIS: ductal carcinoma *in situ*; EDAC: 1-ethyl-3-(3-dimethylaminopropyl)carbodiimide hydrochloride; EPR: enhanced permeability and retention; H&E: hematoxylin and eosin; HSA: human serum albumin; ITTC: indotricarbocyanine; i.v., intravenous; MRI: magnetic resonance imaging; Na: necrotic area; NACA: necrosis avid contrast agent; NHS: N-hydroxysuccinimide; NIR: near infrared; Nv: necrosis volume; PET: positron emission tomography; RFP: red fluorescence protein; RGD: arginine-glycine-aspartic acid; SA: serum albumin; Ta: tumor area; Tv: tumor volume.

## Competing interests

This research is partly funded by grant # BCTR0707388 from 'The Suzan Komen Foundation for the cure', a non-profit organization that may gain some royalties if the discussed novel compounds will be used in the clinics, and Steba-Biotech, France, a pharmaceutical biotech company that develops Bchl-Ds and Bchl-D-RGDs for vascular targeted photodynamic therapy and imaging of solid tumors based on Avigdor Scherz and Yoram Salomon patents. AS and YS have received consultant fees for advising Steba Biotech. The Bchl-Ds and Bchl-D-RGDs disclosed in this manuscript have been patented by 'Yeda' and licensed exclusively to Steba Biotech. 'Yeda' is a non-commercial branch of the Weizmann Institute, Rehovot, Israel, in which AS and YS serve as Professors and group leaders. Vyacheslav Kalchenko is employed as a senior research associate at the Weizmann institute and is in charge of the imaging laboratory. Avigdor Scherz, Yoram Salomon, Liat Goldshaid, Efrat Rubinstein and Alexander Brandis are co-inventors of the used compounds and their applications. Doron Eren, is employed by Steba-Labs, Israel which collaborates with Steba-Biotech, France. Yoseph Salitra and Tamar Yecheskel were employed by this company.

## Authors' contributions

This study is in partial fulfillment of LG's Ph.D. thesis requirements at the Feinberg Graduate School of the Weizmann Institute of Science. LG made major contributions to this manuscript, including all animal experiments, data acquisition and manipulations. ER is co-inventor of the (Bchl-D)-RGD compounds. ER synthesized STL-6009 and demonstrated its accumulation in tumors of different cell lines in partial fulfillment of her Ph.D. thesis at the Weizmann Institute. AB is co-inventor of the STL compounds. AB supervised the *in vitro *fluorescence measurements. DS developed the spectroscopic titration method and supervised its application, as well as binding affinity calculations of the different STL/HSA non-covalent complexes. NL performed part of the spectroscopic titration and binding affinity calculations of the different STL/HSA non-covalent complexes in partial fulfillment of her M.Sc. studies at the Feinberg Graduate School of the Weizmann Institute of Science. In addition, NL conducted part of the biodistribution assays. OB performed the histopathology analysis. VK assisted in planning and supervising the fluorescence imaging. DE up scaled production of the STL compounds. TY up scaled production of the STL compounds. Y. Salitra up scaled production of the STL compounds. Y. Salomon is a group head, co-inventor of the STL compounds and WST11 and co-supervisor of the project. AS is a Group head, co-inventor of the STL compounds and WST11 and supervisor of the project.

## Supplementary Material

Additional file 1 **Optical absorption and fluorescence of compounds used in this study**. Optical absorption and fluorescence in the visible-near infrared domain of the red fluorescence protein and bacteriochlorophyll moieties used in this study. Click here for file

Additional file 2 **Follow-up of STL-6014 accumulation for nine days**. Follow-up of STL-6014 accumulation in orthotopically-grafted large, MDA-MB-231-RFP tumors for nine days. Click here for file

Additional file 3 **STL-6014 accumulation in various body tissues**. STL-6014 accumulation in various body tissues at different time points post injection. Click here for file

Additional file 4 **Changes in RFP fluorescence after STL-6014 administration**. Changes in red fluorescence protein (RFP) fluorescence in large and small tumors after administration of STL-6014. Click here for file

Additional file 5 **STL-6014 accumulation in the necrotic area of large MDA-MB-231-RFP tumors**. STL-6014 accumulation in the necrotic area of large MDA-MB-231-RFP tumors at five and seven days post administration. Click here for file
