# Supplementary material for: Novel design principles enable specific targeting of imaging and therapeutic agents to necrotic domains in breast tumors
Source: Breast Cancer Res. 2010 May 24;12(3):R29. doi: 10.1186/bcr2579 (PMC2917020; doi:10.1186/bcr2579)
Supplement: Additional file 1 — Optical absorption and fluorescence of compounds used in this study. Optical absorption and fluorescence in the visible-near infrared domain of the red fluorescence protein and bacteriochlorophyll moieties used in this study. [file bcr2579-S1.DOC]

**Additional file 1: Optical absorption and fluorescence in the Visible-NIR domain of the RFP and Bchl moieties used in this study.**

**
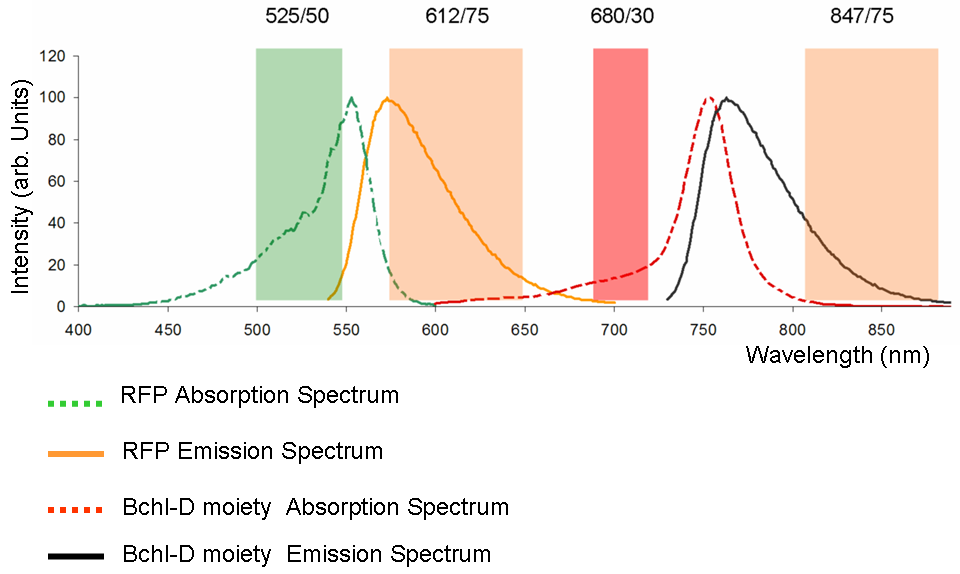
**

The dashed and solid lines stand for the optical absorption and fluorescence of the fluorophores. The bound RGD moieties had minimal effects (± 2nm) on the absorption and fluorescence maxima of the Bchl moiety. The full rectangles represent the light transmittance domains of the filters used in the study.
