# Supplementary material for: Novel design principles enable specific targeting of imaging and therapeutic agents to necrotic domains in breast tumors
Source: Breast Cancer Res. 2010 May 24;12(3):R29. doi: 10.1186/bcr2579 (PMC2917020; doi:10.1186/bcr2579)
Supplement: Additional file 2 — Follow-up of STL-6014 accumulation for nine days. Follow-up of STL-6014 accumulation in orthotopically-grafted large, MDA-MB-231-RFP tumors for nine days. [file bcr2579-S2.DOC]

**Additional file 2: Follow-up of STL-6014** **accumulation** **in orthotopically-grafted large, MDA-MB-231-RFP tumors for 9 days** **
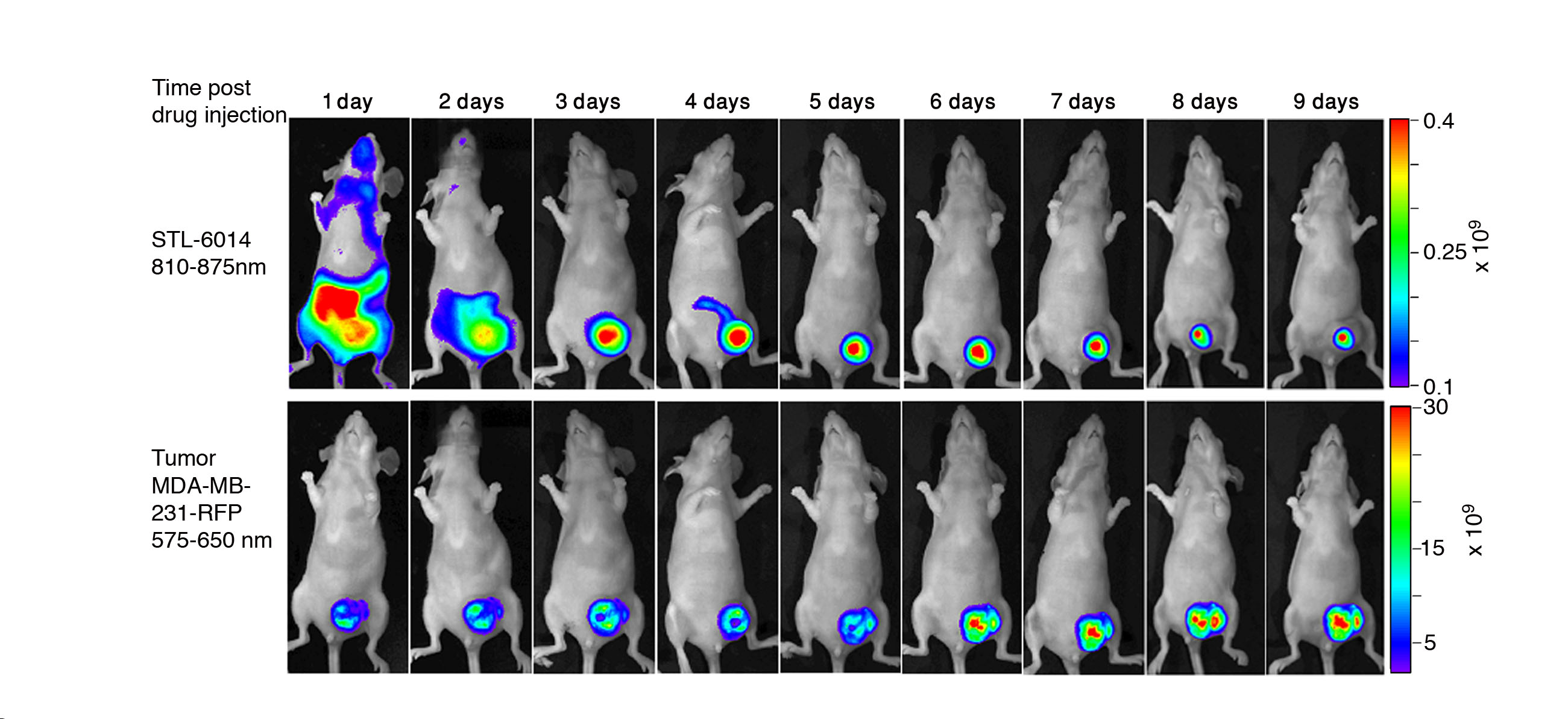
**

CD-1 nude, female MDA-MB-231-RFP tumor-bearing mice (N=9) were i.v. injected with STL-6014 (15 mg/kg). Fluorescent images of large tumors were taken at the indicated times post-injection (1-9 days). Upper panel - NIR fluorescence images indicate STL-6014 distribution, lower panel - red fluorescence images indicate tumor size and location.
