# Supplementary material for: Novel design principles enable specific targeting of imaging and therapeutic agents to necrotic domains in breast tumors
Source: Breast Cancer Res. 2010 May 24;12(3):R29. doi: 10.1186/bcr2579 (PMC2917020; doi:10.1186/bcr2579)
Supplement: Additional file 3 — STL-6014 accumulation in various body tissues. STL-6014 accumulation in various body tissues at different time points post injection. [file bcr2579-S3.DOC]

**Additional file 3: STL-6014** **accumulation in various body tissues
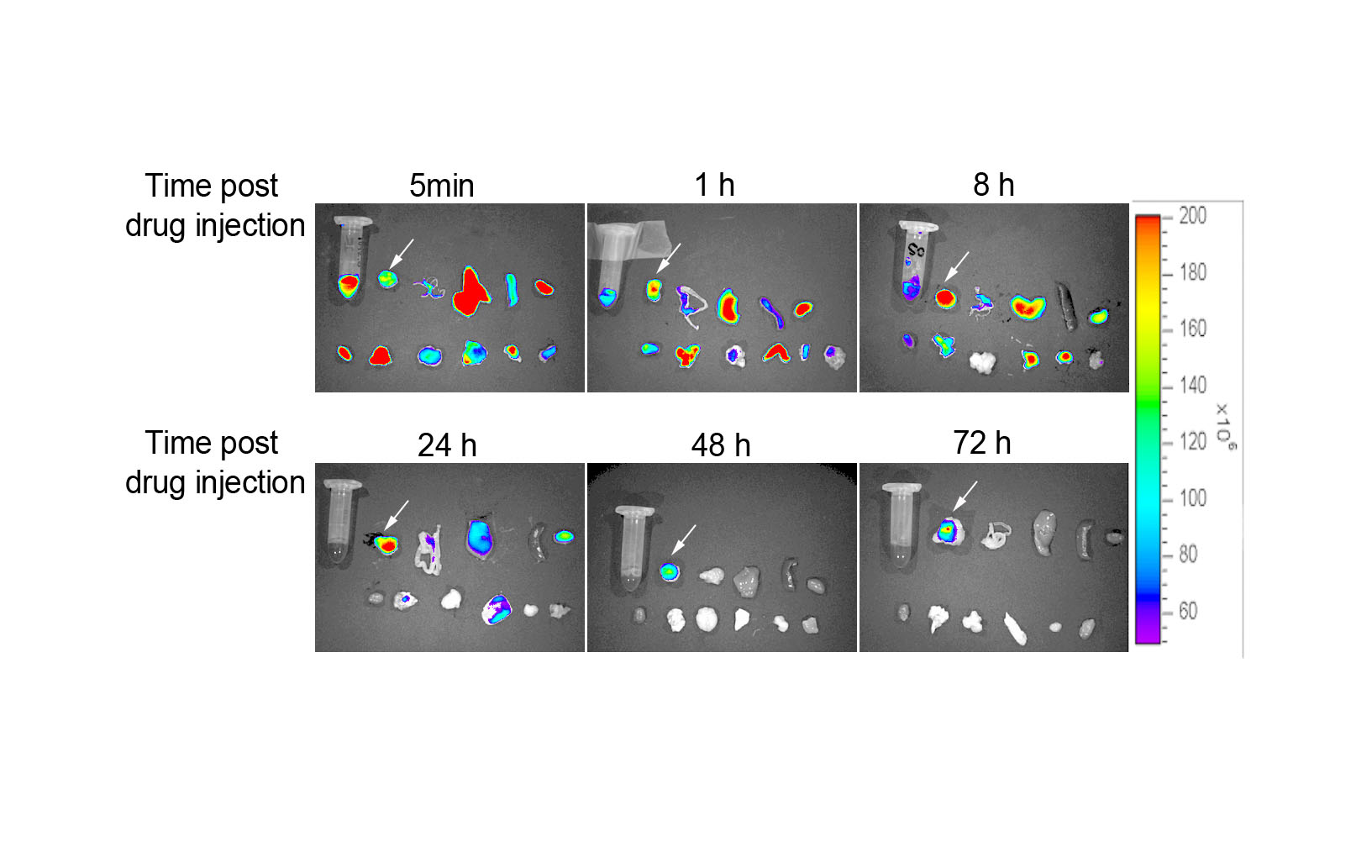
**

CD-1 nude, female, MDA-MB-231-RFP tumor-bearing mice were i.v. injected with STL-6014 (15 mg/kg). Mice (N=3) were sacrificed at the indicated times thereafter, and tissues were collected for fluorescence imaging (810-875 nm). Tissues taken from a representative animal, at each time point are shown. Organs (from left to right); Upper row: blood, tumor, intestine, liver, spleen, kidneys. Lower row: heart, lungs, brain, skin, fat, muscle. Tumor marked with an arrow.
