# Supplementary material for: Novel design principles enable specific targeting of imaging and therapeutic agents to necrotic domains in breast tumors
Source: Breast Cancer Res. 2010 May 24;12(3):R29. doi: 10.1186/bcr2579 (PMC2917020; doi:10.1186/bcr2579)
Supplement: Additional file 4 — Changes in RFP fluorescence after STL-6014 administration. Changes in red fluorescence protein (RFP) fluorescence in large and small tumors after administration of STL-6014. [file bcr2579-S4.DOC]

**Additional file 4:** **Changes in RFP fluorescence in large (A) and small (B) tumors after administration of STL-6014**


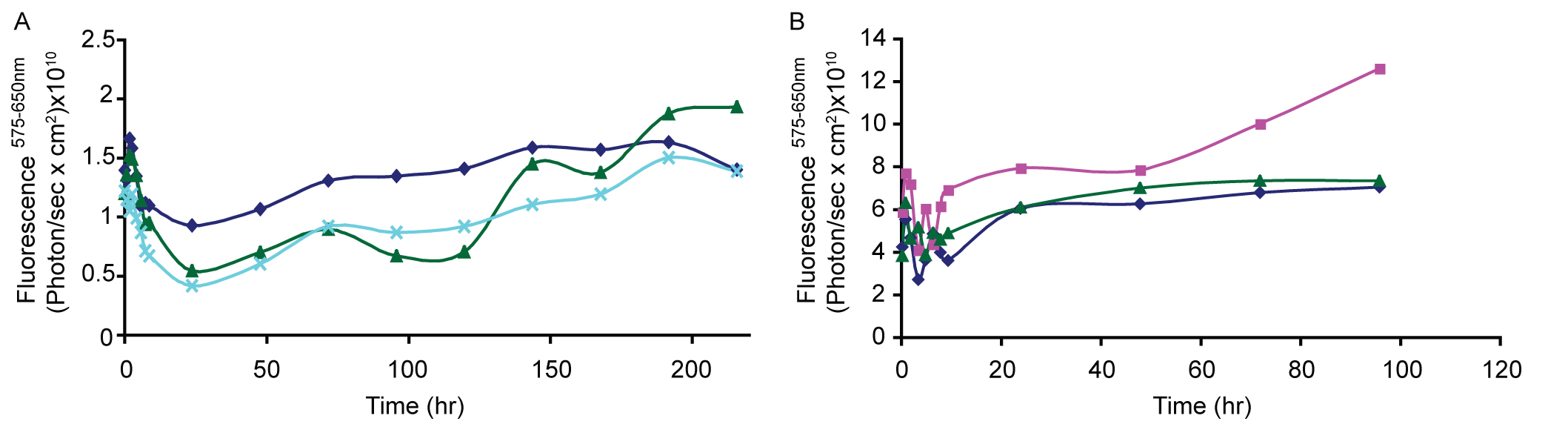


CD-1 nude, female, MDA-MB-231-RFP tumor-bearing mice were i.v. injected with STL-6014 (15 mg/kg). Total RFP fluorescence intensity within the individual tumor boundaries at each time point, was normalized per unit area and expressed as photon/(sec x cm^2^). Each graph represents longitudinal follow-up of one animal (N=3). RFP fluorescence decayed to 50-75% of its initial value within minutes of STL-6014 administration. Fluorescence recovery began at 6-8 h from STL-6014 administration in the small tumors (B) and at ~24 h in large tumors (A). Fluorescence recovery was parallel to STL-6014 clearance from the tumors. As shown in Additional file 1, a small overlap exists between RFP fluorescence and the optical absorption of STL-6014 (600-700nm). Thus, we suggest that the transient reduction in RFP fluorescence reflects its re-absorption by proximal STL-6014 molecules within the viable tumor domain. After STL-6014 clearance from the tumor viable domain, tumor cells regain their RFP fluorescence intensity.
