# Supplementary material for: Novel design principles enable specific targeting of imaging and therapeutic agents to necrotic domains in breast tumors
Source: Breast Cancer Res. 2010 May 24;12(3):R29. doi: 10.1186/bcr2579 (PMC2917020; doi:10.1186/bcr2579)
Supplement: Additional file 5 — STL-6014 accumulation in the necrotic area of large MDA-MB-231-RFP tumors. STL-6014 accumulation in the necrotic area of large MDA-MB-231-RFP tumors at five and seven days post administration. [file bcr2579-S5.DOC]

**Additional file 5 : STL-6014 accumulation in the necrotic area of large MDA-MB-231-RFP tumors**


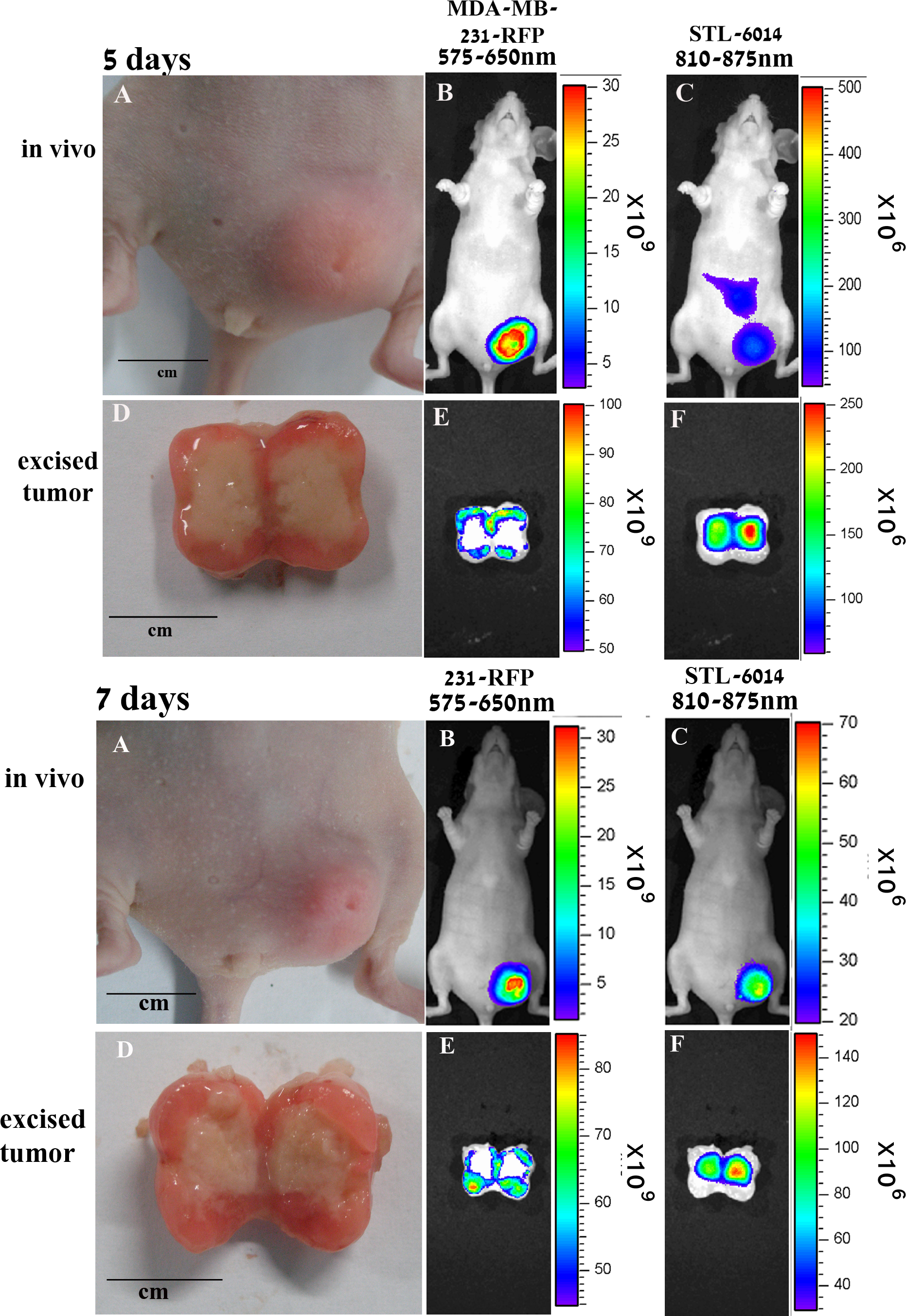


Mice (N = 3) bearing orthotopically-grafted, large MDA-MB-231-RFP tumors were i.v. injected with 15 mg/kg STL-6014. Whole-body and excised tumor images were taken 5 d and 7 d after STL-6014 injection. Upper panels - *in vivo* images, lower panels – images of excised tumors. A&D: color photos. B&E: Red fluorescence images for detection of tumor location and size. C&F: NIR fluorescence images for STL-6014 detection.
